# Altered serum level of metabolic and endothelial factors in patients with systemic sclerosis

**DOI:** 10.1007/s00403-019-01993-y

**Published:** 2019-10-30

**Authors:** Anna Stochmal, Joanna Czuwara, Michał Zaremba, Lidia Rudnicka

**Affiliations:** Department of Dermatology, Medical University of Warsaw, Koszykowa 82A, 02-008 Warsaw, Poland

**Keywords:** Systemic sclerosis, Pathogenesis, Adipose tissue, Endothelial cells, Adiponectin, Resistin, Leptin, Endothelin-1, Fractalkine, Galectin-3

## Abstract

Systemic sclerosis (SSc) is a chronic connective tissue disease characterized by progressive fibrosis, vascular impairment and immune abnormalities. In recent years, adipokines (mediators synthetized by adipose tissue) have been indicated as a possible missing link in the pathogenesis of SSc. The aim of this study was to investigate the serum concentration of metabolic adipose tissue factors: adiponectin, resistin, leptin and endothelial proteins: endothelin-1, fractalkine and galectin-3 in patients with systemic sclerosis. The study included 100 patients with confirmed SSc diagnosis and 20 healthy individuals. The concentration of respective proteins was determined by enzyme-linked immunosorbent assay. The following markers showed statistically significant increased mean concentrations in patients with SSc in comparison to healthy control: resistin (13.41 vs 8.54 ng/mL; *P* = 0.0012), endothelin-1 (1.99 vs 1.31 pg/mL; *P* = 0.0072) and fractalkine (2.93 vs 1.68 ng/mL; *P* = 0.0007). Elevated serum levels of galectin-3 (4.54 vs 3.26 ng/mL; *P* = 0.0672) and leptin (19,542 vs 14,210 pg/mL; *P* = 0.1817) were observed. Decreased concentration of adiponectin was found in patients with SSc (5150 vs 8847 pg/mL; *P* = 0.0001). Fractalkine and galectin-3 levels were significantly higher in diffuse cutaneous SSc than limited cutaneous SSc subset (3.93 ng/mL vs 2.58 ng/mL, *P* = 0.0018; 6.86 ng/mL vs 3.78 ng/mL, *P* = 0.0008, respectively) and correlated positively with modified Rodnan Skin Score in total SSc patients (*r* = 0.376, *P* = 0.0009; *r* = 0.236, *P* = 0.018, respectively). In conclusion, an increased serum level of resistin associated with increased endothelin-1 and fractalkine level and decreased adiponectin level may indicate a significant role of the adipose tissue in the development and progression of vascular abnormalities in patients with systemic sclerosis. Fractalkine and galectin-3 may participate in promoting and exacerbating the fibrotic process in SSc.

## Introduction

Systemic sclerosis (SSc) is a chronic connective tissue disease characterized by progressive fibrosis along with vascular impairment and immune abnormalities [1, 2]. Adipokines, including adiponectin, resistin and leptin, consist a group of molecules synthesized mainly by adipose tissue cells. They possess a pleiotropic effect on many metabolic pathways and participate in the development of autoimmune and inflammatory diseases, embracing SSc [3]. Adiponectin is a plasma protein with anti-inflammatory and anti-fibrotic activity which inhibits production of proinflammatory cytokines such as TNF-α and IL-6 [4]. Resistin is a polypeptide synthetized in abundance also by immune cells and has properties contradictory to adiponectin, induces inflammation and activates the differentiation of regulatory T lymphocytes which produce and secrete TGF-β—a key profibrotic cytokine [5]. Leptin was primarily described as a molecule involved in appetite regulation and energy homeostasis [6]. However, it also plays an important role in both innate and adaptive immunity promoting phagocytosis, Th-1 lymphocytes activation and inflammation, mostly by stimulating monocytes to release pro-inflammatory cytokines [7].

In addition to adipokines, it has been reported that molecules released by endothelial cells, such as endothelin-1, fractalkine and galectin-3, are involved in pathological process in SSc [8]. Endothelin-1, a predominant one of three isoforms in humans, is considered a pivotal element in vasoconstriction regulation [9]. Moreover, this peptide exerts a complex fibrogenic effect: directly affects fibroblasts and indirectly stimulates fibrosis by enhancing TGF-β effect [10]. Another molecule which is perceived to participate in endothelial injury is fractalkine. Two forms of fractalkine may be distinguished: a soluble in serum and bound with a membrane of endothelial cells. Both exhibit a potent chemotactic activity for lymphocytes T, monocytes and natural killer cells [11]. Furthermore, membrane-linked form mediates immune cells adhesion and migration through the vessel wall resulting in perivascular infiltration of inflammatory cells, which is an event preceding the development of fibrosis [12]. Structural changes of blood vessels are mediated also by galectin-3—a carbohydrate-binding protein expressed and secreted to the circulation by the variety of cells, mostly endothelial and immune cells. It is involved in many biological processes, such as fibroblast activation and fibrosis induction following tissue damage, cell adhesion and angiogenesis [13].

Taking into consideration essential functions of mediators derived from adipose tissue and endothelial cells physiological and pathological processes, every disharmony in their production or activity may contribute to the development of the disease [14, 15]. The previous studies showed ambiguous results concerning measurement of the above mentioned molecules in sera of patients with SSc, probably due to small groups and their demographic heterogeneity [16]. The aim of this study was to investigate the concentrations of the serum markers potentially associated with the pathogenesis and clinical course of the disease in a representative group of SSc-patients. To the best of the knowledge, this is the first study of simultaneous evaluation of metabolic and endothelial factors combination in sera of patients with SSc.

## Patients and methods

### Patients

The study included 100 patients with SSc fulfilling the 2013 ACR/EULAR Classification Criteria for Systemic Sclerosis [17] [74 patients with limited cutaneous systemic sclerosis (lcSSc) and 26 patients with diffuse cutaneous systemic sclerosis (dcSSc); 94 women, 6 men; mean age 58.3 years (range 30–89)] and 20 healthy individuals matched for age, sex and body mass index. Severity of skin fibrosis was evaluated by modified Rodnan Skin Score (mRRS) [18]: mean 8.27 (range 2–30) in total SSc patients, mean 5.31 (range 2–10) in lcSSc subset, mean 16.69 (range 9–30) in dcSSc subset of the disease. All hospitalized patients were receiving cyclic intravenous rheological treatment with either prostacyclin analogue or sulodexide, while in ambulatory treatment oral vasoactive medications as calcium channel blockers, sildenafil and/or sulodexide were administered. A part of SSc patients, in particular those with exacerbated fibrotic process were treated with metothrexate up to 20 mg per week or mycophenolate mofetil up to 3 g per day.

Patients who met any of the following criteria were excluded from the study: diagnosis of overlap syndrome, age < 18 years and pregnancy. The study protocol was conformed to the principles of the World Medical Association’s Declaration of Helsinki and was approved by the local ethics committee. The written informed consent was obtained from all participants of the study.

### Methods

All blood samples (6 mL each) were collected to obtain serum for further analysis. The frozen serum samples were stored at − 80 °C until the analysis. To assess metabolic disturbances, serum concentrations of adiponectin, resistin and leptin were measured. To assess vascular and endothelial abnormalities, concentrations of endothelin-1, fractalkine and galectin-3 were measured. The concentrations of all proteins were assessed by enzyme-linked immunosorbent assays (Quantikine ELISA Kits R&D Systems, Minneapolis, USA) according to the protocol provided by manufacturer.

### Statistical analysis

The data distributions were assessed for normality with Shapiro–Wilk test. To compare differences between groups, Student’s *t* test for data with normal distribution and Mann–Whitney *U* test for non-parametric variables was used. Spearman’s rank test was used to measure correlations between variables (*r*). Data were considered significant for *P* < 0.05. Analyses were performed with SAS version 9.4 software.

## Results

**Fig. 1:**
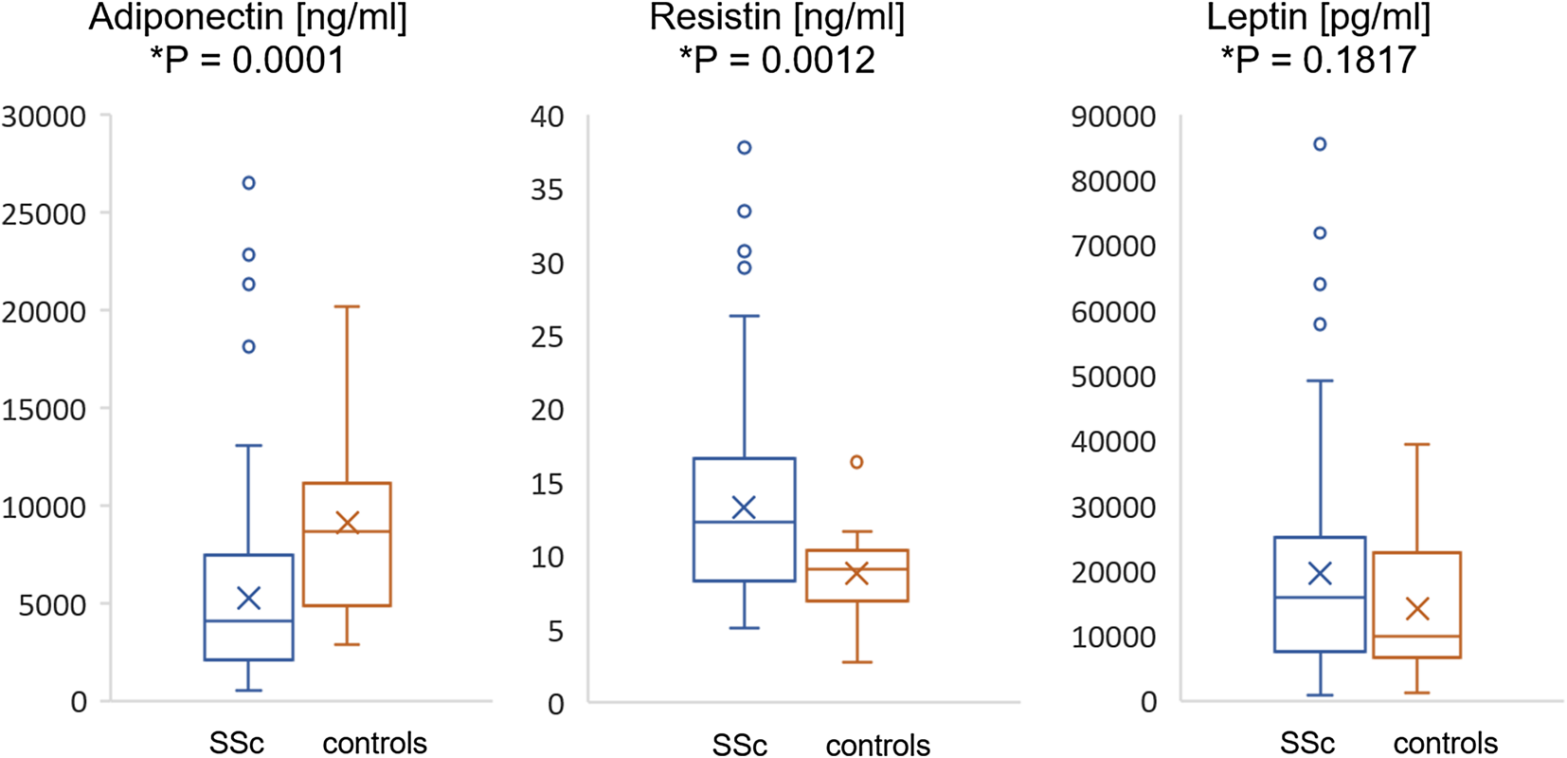
Serum concentration of adiponectin, resistin and leptin in SSc vs. healthy controls. Results presented as median and interquartile range

**Fig. 2:**
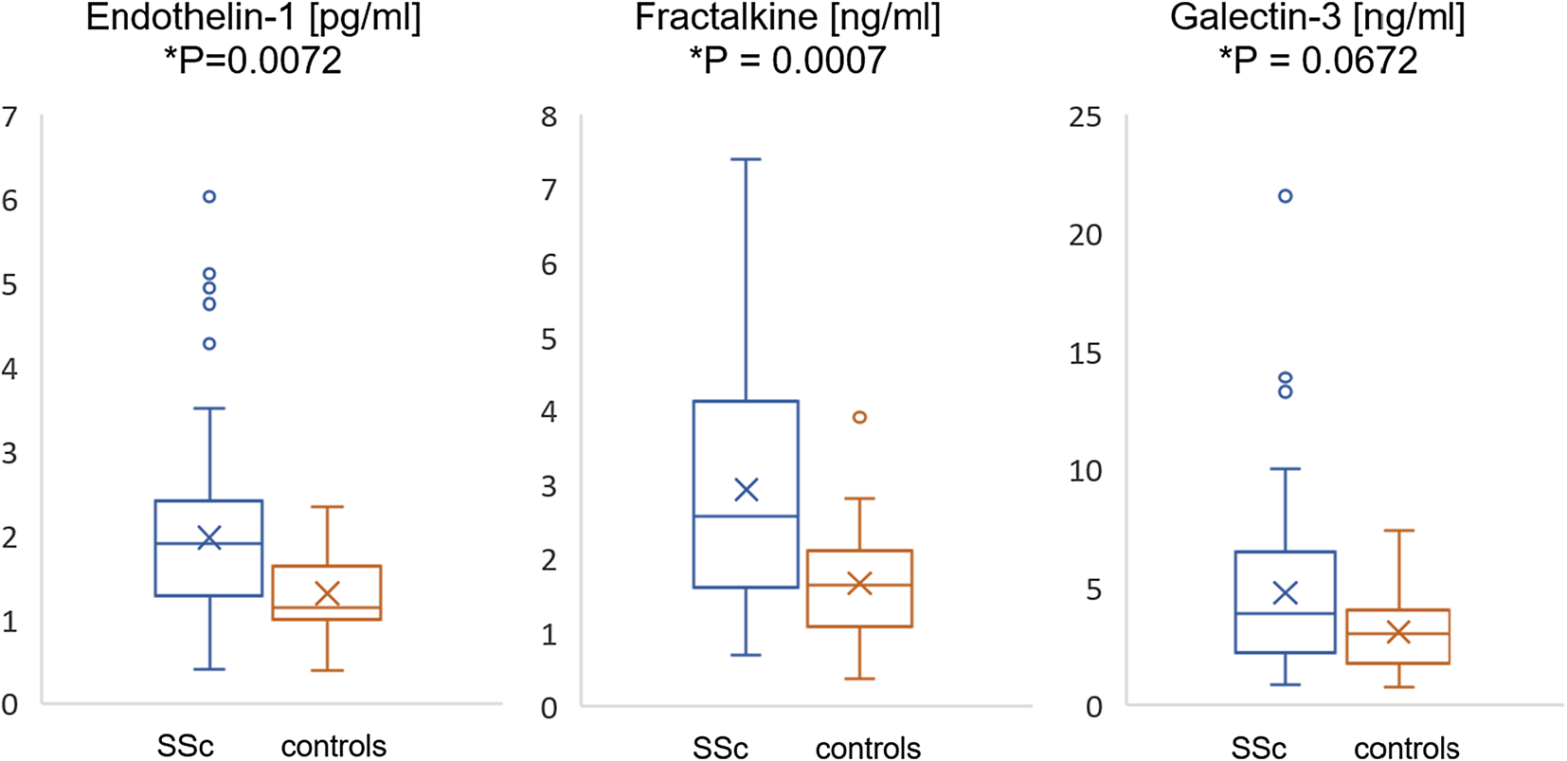
Serum concentration of endothelin-1, fractalkine and galectin-3 in SSc vs. healthy controls. Results are presented as median and interquartile range

Mean concentrations of resistin [13.41 ng/mL; (range 5.11–37.75) vs 8.54 ng/mL (range 3.54–16.39); *P* = 0.0012], endothelin-1 [1.99 pg/mL; (range 0.51–6.03) vs 1.31 pg/mL (range 0.40–2.34); *P* = 0.0072] and fractalkine [2.93 ng/mL; (range 0.69–6.99) vs 1.68 ng/mL (range 0.37–3.91); *P* = 0.0007] were significantly higher in sera of total SSc patients than in the healthy control. Moreover, serum levels of galectin-3 [4.54 ng/mL; (range 0.85–21.57) vs 3.26 ng/mL (range 0.74–7.43); *P* = 0.0672] and leptin [19,542 pg/mL; (range 1041–85,500) vs 14,210 pg/mL (range 1286–39,480); *P* = 0.1817] were elevated in SSc patients. Concentration of adiponectin was found to be significantly decreased in the SSc studied group [5150 ng/mL; (range 546–26,500) vs 8847 ng/mL (range 5081–20,160); *P* = 0.0001] (Figs. 1, 2). Comparing concentrations of each factor in particular subsets of SSc, serum fractalkine and galectin-3 levels were statistically significantly higher in dcSSc than lcSSc (3.93 ng/mL vs 2.58 ng/mL, *P* = 0.0018; 6.86 ng/mL vs 3.78 ng/mL, *P* = 0.0008, respectively). Concentrations of other investigated proteins did not show significant differences between SSc subsets (Table 1). Weak but significant positive correlation with mRRS and serum level of fractalkine (*r* = 0.376, *P* = 0.0009) and galectin-3 (*r* = 0.236, *P* = 0.018) was indicated. Correlations of another studied proteins with mRRS were not statistically significant.

**Table 1:** Serum concentration of metabolic and endothelial factors in patients with lcSSc and dcSSc

Mean $\pm$ SD	lcSSc (*n* = 74)	dcSSc (*n* = 26)	P-value
Adiponectin (ng/mL)	5244 $\pm$ 4833	4515 $\pm$ 4387	0.3014
Fractalkine (ng/mL)	2.58 $\pm$ 1.38	3.93 $\pm$ 1.38	0.0018
Resistin (ng/mL)	12.21 $\pm$ 5.98	15.60 $\pm$ 8.04	0.0707
Endothelin-1 (pg/mL)	1.81 $\pm$ 1.04	2.03 $\pm$ 1.24	0.6914
Galectin-3 (ng/mL)	3.78 $\pm$ 2.51	6.86 $\pm$ 4.76	0.0008
Leptin (pg/mL)	18,062 $\pm$ 14,864	18,510 $\pm$ 19,992	0.7060

*lcSSc *limited cutaneous systemic sclerosis, *dcSSc* diffuse cutaneous systemic sclerosis, *SD* standard deviation

## Discussion

In recent years adipokines, molecules synthetized by adipose tissue involved in metabolism and several signaling proteins secreted by endothelial cells were suggested in the development of SSc [2]. Therefore, measurement of the concentration of these circulating biomarkers may be of great clinical importance. The study presents altered serum levels of particular molecules that may have an impact on the pathogenesis of SSc.

In this study, a statistically significant increased concentration of resistin was found in patients with SSc. It complies with the elevated serum resistin level in other chronic systemic inflammatory diseases, including rheumatoid arthritis and systemic lupus erythematosus [19, 20]. Taking into consideration biological profile of action, this observation suggests that increased level of resistin results in severe inflammation which seems to play one of the major roles in the pathogenesis of SSc. Interestingly, it was reported in the previous studies that resistin directly activates endothelial cells by stimulating endothelin-1 release [21]. It is consistent with the results of this study as an increased concentration of endothelin-1 was indicated in SSc patients. This may confirm the hypothesis that impaired level of adipose tissue factors influences also endothelial dysfunction and aberrant angiogenesis which is one of the main features of SSc. The excess of circulating endothelin-1 may be the cause of micro- and macrovascular changes and their further complications such as digital ulcers formation and pulmonary arterial hypertension frequently observed in SSc patients [22]. It should be also emphasized that an initial vascular injury results in an exacerbated inflammatory response which finally promotes fibrosis [23]. The endothelial damage leading to exacerbated fibrosis may be also caused by fractalkine [24] which is significantly elevated in sera of patients with SSc, in particular in dcSSc subset, as the current research has shown. It is consistent with positive correlation between fractalkine along with galectin-3 and severity of cutaneous fibrosis measured by mRRS. Higher serum level of galectin-3 was also observed in SSc patients, although the difference in the concentration between SSc group and healthy controls was not as significantly marked as in other endothelial factors in this study. However, in the present research galectin-3 level showed significant preponderance in dcSSc compared to lcSSc subset, which may confirm the assumption that galectin-3 is related to the exacerbation of fibrosis and depends on the stage of the disease as it was previously described [25]. Current data on the concentration of leptin in SSc are ambiguous and either elevated, decreased or comparable levels to healthy control have been reported [16, 26]. In the present study serum levels of leptin were higher than in the control group, although the difference did not reach statistical significance. These discrepancies among studies may be the result of a different duration or the activity of the disease, as it was suggested in the literature [27]. Higher levels of leptin may imply in constant inflammation of various intensity which is pronounced especially in an active phase of SSc and due to this finding leptin was suggested as an activity marker in SSc [27].

The present research showed also that the concentration of adiponectin in SSc patients was significantly decreased in comparison to healthy individuals. With regard to the anti-fibrotic and anti-inflammatory action of adiponectin, the deficiency of this molecule may contribute to the development of fibrosis and triggering immune response [28].

To conclude, alterations in the concentration of each factor examined in this study may disturb the variety of metabolic and vascular processes, leading to local and systemic immune response and intense fibrosis. In particular, increased serum resistin associated with increased endothelin-1 and fractalkine level and decreased adiponectin level may indicate that adipose tissue participates in the development and progression of vascular, inflammatory and fibrotic abnormalities in patients with systemic sclerosis. Moreover, endothelial factors including especially fractalkine and galectin-3 may be relevant in exacerbating severity of fibrosis in SSc. The results of this research support the hypothesis that altered levels of both adipokines and endothelial factors may play an important and complex role in SSc.
